# Advancing qualitative rare disease research methodology: a comparison of virtual and in-person focus group formats

**DOI:** 10.1186/s13023-022-02522-3

**Published:** 2022-09-11

**Authors:** Andrew A. Dwyer, Melissa Uveges, Samantha Dockray, Neil Smith

**Affiliations:** 1 William F. Connell School of Nursing, Boston College, Chestnut Hill, MA USA; 2 Massachusetts General Hospital-Harvard Center for Reproductive Medicine, Boston, MA USA; 3 School of Applied Psychology, University College Cork, Cork, Ireland; 4 HYPOHH Patient Support Group, London, UK

**Keywords:** Community based participatory research, Genetic testing, Hypogonadotropic hypogonadism, Kallmann syndrome, Qualitative research methods, Rare disease

## Abstract

**Background:**

Rare disease research is hampered in part by the fact that patients are geographically dispersed. Rare disease patient communities are recognized for their use of the internet to learn about their condition and find peer-to-peer support. As such, web-based technologies offer promise for overcoming geographic barriers in rare disease research for many. Qualitative focus groups (FGs) are a widely used methodology used to understand patients and parents/families ‘lived experience’ and unmet needs is important to improve care for rare diseases. It is unclear if web-enabled (virtual) FGs are comparable to traditional in-person approaches. We conducted in-person (n = 3) and virtual (n = 3) FGs with rare disease patients to determine if virtual FGs produce similar results in-person FGs.

**Results:**

Three in-person (n = 33 participants) and three virtual (n = 25 participants) FGs were conducted examining attitudes and beliefs regarding genetic testing and family communication of risk. Participants included 30 males, 18 females, and 10 parents/guardians. Two independent investigators identified excerpts (meaningful sections of text) and coded themes/sub-themes using a codebook. Inter-coder agreement across identified excerpts (n = 530) in both FG formats was 844/875 (96.5%). Two additional investigators reviewed coded excerpts and did not identify additional themes/sub-themes—supporting data saturation across FG formats. Virtual FGs accounted for 303/530 (57.2%) of total excerpts and 957/1721 (55.7%) of all identified themes/sub-themes. Formats were similar in terms of overall number of excerpts (101 ± 7.8 vs. 75.7 ± 18.8, *p* = 0.26) and themes/sub-themes (319 ± 6.1 vs. 254.7 ± 103.6, *p* = 0.34) between virtual and in-person FGs. However, virtual FGs had significantly more coded excerpts specifically relating to sensitive/intimate topics including ‘attitudes and beliefs’ (n = 320 vs. n = 235, *p* < 0.001), ‘information and support’ (n = 184 vs. n = 99, *p* < 0.001), and ‘family communication’ (n = 208 vs. n = 114, *p* < 0.001).

**Conclusions:**

Virtual FGs yielded similar numbers of coded excerpts compared to traditional in-person FGs. Virtual FGs appear to support the relative anonymity of participants, resulting in richer discussion of highly sensitive, intimate topics. Findings support the validity and methodologic rigor of using web-enabled technologies for conducting FGs in rare diseases.

**Supplementary Information:**

The online version contains supplementary material available at 10.1186/s13023-022-02522-3.

## Background

A major barrier to conducting rare disease research is that patients with rare diseases are geographically dispersed [1, 2]. Indeed, rare disease studies are typically limited by small sample size (i.e., single-center cohorts), most do not have sufficient power, and many studies are not completed [3]. One approach to overcome such barriers has been to form networks and consortia to aggregate patients and consolidate research on rare diseases [4, 5]. However, there are more than 6000 rare disorders and research networks do not exist for many rare conditions.

In addition to recruitment challenges, geographic barriers also contribute to feelings of isolation and marginalization frequently reported by rare disease patients [6, 7]. For many rare disease patients and parents/families, the internet has been a powerful tool for finding information, locating online patient organizations, and obtaining peer-to-peer support [8]. Researchers have used the internet to enhance enrollment in registries [9] and bolster recruitment for rare disease clinical trials [10, 11]. Some investigators collaborate with advocacy groups (i.e., patient support organizations) to overcome recruitment barriers [12, 13]. Others have embraced less transactional models of research by actively engaging patients as key stakeholders and partners in research (i.e. community-based participatory methods) thereby enhancing patient recruitment [14].

Rare disease patients have been referred to as internet “power-users” due to their facile use of the internet [15]. We have previously validated using community engagement with patient organizations and online recruitment to reach geographically dispersed rare disease patients for quantitative research [16]. However, it is unclear if using web-based technologies for qualitative rare disease research (e.g., interviews, focus groups) produce similar results as traditional, in-person methods.

In this study, we aimed to compare traditional face-to-face and web-enabled (virtual) qualitative research using focus groups (FGs). Given the well-recognized geographic barriers to rare disease research, we considered that demonstrating validity of virtual FGs (compared to traditional in-person methods) could support methodologic rigor of qualitative inquiry in rare diseases and hold implications for the broader rare disease research community.

## Methods

The prospective qualitative study involved both in-person (n = 3 face-to-face) and virtual (n = 3 online) focus group discussions. The study was conducted in accordance with the Declaration of Helsinki. The study protocol was reviewed and approved by the ethics committee of the University of Lausanne (protocol #2016_02184) and the Boston College Institutional Review Board (protocol #18.081.01). Participants provided written informed consent or opt-in electronic consent for in-person and virtual focus groups respectively and were offered a $25 gift card as remuneration for study participation.

### Participants

Focus group participants included adult patients and parents/guardians of children with congenital hypogonadotropic hypogonadism (CHH, ORPHA174590)/ Kallmann syndrome (KS, ORPHA478). Briefly, CHH/KS is a rare endocrine disorder resulting from deficient secretion (or action) of gonadotropin releasing hormone (GnRH) that clinically manifests as absent/incomplete puberty and infertility [17]. There are a range of non-reproductive phenotypes associated with CHH, most notably about half of patients have impaired olfactory function (anosmia)—a feature that differentiates normosmic CHH from KS [17]. Other associated phenotypes occur at variable rates including midline defects (i.e., cleft lip/palate), skeletal/dental anomalies, unilateral renal agenesis, synkinesia (mirror movements), and sensory deficits (i.e., hearing loss, eye movement disorders) [17]. By current estimates, CHH occurs in approximately 1:48,000 persons [18] with a notable 4:1 (male:female) sex discordance [17].

Unlike many rare diseases, effective treatments are available for inducing secondary sex characteristics (i.e., sex steroid replacement) and specialized treatments (i.e., gonadotropin injections or pulsatile GnRH) can induce fertility in roughly 75–80% of cases [17]). Notably, CHH/Ks is a diagnosis of exclusion and delayed diagnosis is common [19, 20]. Moreover, later diagnosis is associated with increased psychosocial morbidity as well as psychosexual concerns [16, 21, 22]. Similar to the variability in clinical presentation, CHH/KS is genetically heterogeneous. To date, more than 60 loci has been identified to underlie CHH/KS, accounting for approximately half of cases [23]. Inheritance patterns include X-linked, autosomal recessive, and autosomal dominant yet many cases are characterized by incomplete penetrance and variable expressivity [23]. Further, the genetic architecture is complex with digenic and oligogenic cases having been reported [23].

To recruit the purposive sample, we employed a community-based participatory research framework [24, 25]. We partnered with a CHH/KS patient community leader (NS) to co-organize and conduct informational patient meetings (in-person and virtual). Patient meetings provide general information about CHH/KS including an overview of pathophysiology, genetics, diagnosis, treatment options, living with CHH/KS, and health promotion topics. At the close of the informational meetings, English-speaking adult patients (18-years and older) and parents/guardians of a child/adolescent with CHH/KS were invited to participate in a focus group discussion.

### Focus group (FG) discussions

The face-to-face FGs were conducted at annual in-person patient meetings (2017–2019). Virtual FGs were conducted over Zoom (January to June 2021). Semi-structured FG discussions (90–120 min) were led by an investigator (AD) and the patient group leader (NS). The question prompts related to experiences around genetic testing for CHH/KS, issues related to decision-making, and discussing CHH/KS with potentially at-risk blood relatives (Additional file 1). All FG discussions were audio recorded and transcribed verbatim. Transcripts were deidentified (i.e., assigning participant numbers) for qualitative analysis (coding). A critical aspect of qualitative inquiry is the concept of saturation—meaning the time at which no new codes emerge. Typically, three to five FGs discussions are needed to reach saturation [26]. We have previously demonstrated that saturation can be reached in three FGs with rare disease patients (i.e., CHH/KS) [27]. We conducted three in-person and three virtual FGs to reach data saturation for each approach to ensure an appropriate comparison of methods (i.e. in-person vs. virtual).

### Analyses

We employed thematic analysis for coding FG discussions as previously described [27]. To create the codebook for analyzing FG transcripts, two independent investigators (AD, MU) reviewed and coded the first face-to-face FG transcript using Dedoose (Version 9.0.17, SocioCultural Research Consultants LLC, Los Angeles, CA, www.dedoose.com). Briefly, investigators highlighted excerpts (sections of meaningful content in the transcript) and labeled these with an identifying code (representing a theme or sub-theme). Subsequently, investigators met to create the codebook by discussing themes/sub-themes, collapsing similar codes and operationalizing theme/sub-theme definitions. Differences in coding the initial FG transcript were resolved by discussion to create the codebook. Investigators then used the codebook to independently code the remaining five transcripts for themes/sub-themes. Following completion of consensus coding, focus group transcripts and the list of themes/sub-themes were sent to two additional investigators who served as external reviewers to validate (i.e., ensure all themes/subthemes were appropriately captured) the consensus coding. The patient group leader (NS) and an investigator experienced in qualitative research (SD) served as reviewers to validate the consensus coding.

Participant characteristics are reported using descriptive statistics (i.e., range, median, mean, standard deviation). Codes (i.e., themes/sub-themes) were mapped for presence in each FG discussion (in-person vs. virtual). Inter-coder reliability was determined for both highlighted excerpts and codes. The number of excerpts and codes (themes/sub-themes) appearing in FG discussions were compared to in-person FG #1 excerpt/codes (used to create the codebook). Categorical between-group comparisons were performed using Chi-square tests. Student’s T-test was used to compare the number of identified excerpts and codes between in-person and virtual FGs. A *p* value < 0.05 was considered statistically significant.

## Results

Six FG discussions were conducted including three in-person (n = 33 participants) and three virtual (n = 25 participants). Overall, the purposive sample included 30 males, 18 females, and 10 parents/guardians. Participant characteristics are depicted in Table 1. Rates of male patients and parents/guardians were similar between in-person and virtual FGs. Virtual FGs included more female participants (n = 12 vs. n = 6, *p* = 0.032). This difference is accounted for by the fact that female patients asked the patient support organization for a dedicated “female only” meeting. Male and female participants were similarly aged between in-person and virtual FGs yet the parents/guardians in the virtual meetings were significantly younger than in-person FGs (31 ± 1.4 years vs. 49 ± 9.9 years, *p* = 0.04).

**Table 1:** Characteristics of focus group participants (n = 58)

	In-person (n = 33)	Virtual (n = 25)	Total (n = 58)
*Male patients (n)*	19	11	30
Age range (years)	20–72	22–75	20–75
Median age (years)	37.0	60.0	40.5
Mean ± SD	39.7 ± 14.4	53.4 ± 18.2*	44.7 ± 17.0
*Female patients (n)* †	6	12*	18
Age range (years)	24–68	33–52	24–68
Median age (years)	33.0	43.0	40.0
Mean ± SD	37.5 ± 16.2	42.8 ± 7.5	40.7 ± 11.5
*Parents/guardians (n)*	8	2	10
Age range (years)	35–62	30–32	30–62
Median age (years)	47.5	31*	45.0
Mean ± SD	49.0 ± 9.9	-	45.4 ± 11.6

SD: standard deviation, *p < 0.05 versus in-person, † one virtual focus group was “female only”

Qualitative analysis revealed five emergent themes and 16 sub-themes in FG discussions (Table 2). The five overarching themes appeared in all FGs. In total, 13/16 (81%) sub-themes appeared in all FGs (Additional file 2). Virtual and in-person FGs were equivalent in terms of presence of themes/sub-themes (i.e., 3 FGs X 21 themes/sub-themes = 63 possible mentions) and 60/63 (95%) of possible themes/sub-themes appeared in both formats. Of the three absent sub-themes, two related to ‘ethical concerns’—the least noted of the six overarching themes (Additional file 2). The ‘ethical concerns’ sub-themes of ‘informed consent’ and ‘sample traceability’ were absent in in-person #3/virtual #1 and in-person#3/virtual #1/virtual#2 respectively. The sub-theme ‘cost’ did not appear in the in-person #2 FG.

**Table 2:** Themes and sub-themes appearing in the in-person (33 participants) and virtual (25 participants) focus groups

Theme	In-person (n = 3)	Virtual (n = 3)	Total (n = 6)
Sub-theme			
*Attitudes and beliefs (n, %)*	106 (43%)	143 (57%) a	249 (100%)
Motivating factors	84 (41%)	120 (59%) b	204 (100%)
Test type	23 (46%)	27 (54%)	50 (100%)
Uncertainty	18 (58%)	13 (42%)	31 (100%)
Cost of testing	4 (19%)	17 (81%) b	21 (100%)
*Information and support (n, %)*	47 (36%)	84 (64%) b	131 (100%)
Information source	20 (24%)	62 (76%) b	82 (100%)
Pre-test decision support	18 (45%)	22 (5%)	40 (100%)
Genetic counseling	14 (47%)	16 (53%)	30 (100%)
*Return of results (n, %)*	79 (48%)	84 (52%)	163 (100%)
Uncertainty	38 (45%)	47 (55%)	85 (100%)
Results interpretation	36 (54%)	31 (46%)	67 (100%)
Lack of results, waiting	37 (69%) b	17 (31%)	54 (100%)
Lack of post-test support	12 (32%)	25 (68%) c	37 (100%)
*Family communication (n, %)*	51 (34%)	97 (66%) b	148 (100%)
Barriers	39 (36%)	70 (64%) b	109 (100%)
Promoters	24 (37%)	41 (63%) c	65 (100%)
*Ethical concerns (n, %)*	46 (70%) b	20 (30%)	66 (100%)
Privacy and data use	29 (66%) d	15 (34%)	44 (100%)
Sample traceability	22 (88%) b	3 (12%)	25 (100%)
Informed consent	17 (85%) b	3 (15%)	20 (100%)

a: *p* = 0.001; b: *p* < 0.001; c: *p* = 0.005; d: *p* = 0.006

A comparison of relative contribution of in-person and virtual FG to each theme/sub-theme is presented in Table 2. Virtual FGs accounted for a significantly greater number of collapsed codes for ‘attitudes and beliefs (n = 320 vs. n = 235, *p* < 0.001), ‘information and support’ (n = 184 vs. n = 99, *p* < 0.001), and ‘family communication’ (n = 208 vs. n = 114, *p* < 0.001). Virtual and in-person FGs had similar numbers of codes for ‘return of results’ (virtual: n = 204, in-person: n = 202, *p* = 0.94). In-person FGs had a greater number of codes for ‘ethical concerns’ (n = 114 vs. n = 41, *p* < 0.001). Thus, while themes nor sub-themes equally present in in-person and virtual FGs, differences were noted in the weighting of contributions to specific themes and sub-themes.

### Inter-coder reliability of independent coding

Thematic analysis of FG discussions yielded a total of 530 excerpts (i.e., highlighted sections of meaningful text) identified by the two independent coders. No differences were observed in the number of excerpts identified by the coders (n = 272 [51.3%] vs. n = 258 [48.7%], *p* = 0.39). In total, 503/530 (94.9%) of excerpts were identified by both coders supporting the fidelity of independent excerpt identification. Each excerpt was labelled with a code reflecting an overarching theme and/or sub-theme. Excerpts were labelled with more than one theme/sub-theme as appropriate. The codebook (composed of 5 themes and 16 subthemes) was applied by independent coders to identify 875 total themes/sub-themes. No differences were observed in the number of codes independently identified by coders (n = 445 [50.7%] vs. n = 430 [49.1%], *p* = 0.29). In total, 844/875 (96.5%) of themes/sub-themes were identified by both coders supporting high concordance (inter-coder reliability) of independent coding. Following independent coding of the FG transcripts, two additional investigators served as external reviewers to review the transcripts and code book. External reviewers verified the consensus coding and did not identify any new themes/sub-themes—confirming that data saturation had been met in both in-person and virtual FGs.

### Comparison of focus group formats

**Fig. 1:**
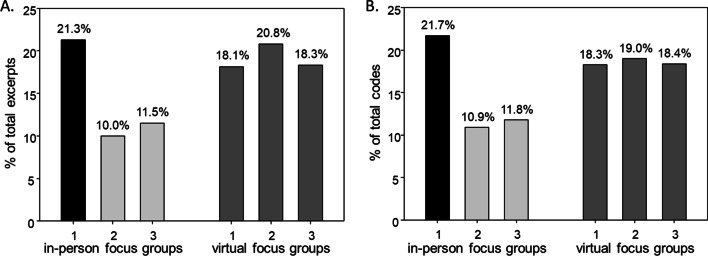
Percentage of total excerpts and codes by focus group. The first in-person FG (black bar) was used to create the code book and was set as the ‘standard’. **A** Excerpts from in person FGs (n = 113, 53, 61 respectively) did not differ from virtual FGs (n = 96, 110, 97 respectively). **B** Codes from in-person FGs (n = 374, 188, 202 respectively) did not differ from virtual FGs (n = 315, 326, 316 respectively)

Virtual FGs accounted for 303/530 (57.2%) of total excerpts. No differences were observed in mean number of excerpts between virtual and in-person FGs (101 ± 7.8 vs. 75.7 ± 18.8, *p* = 0.26). Virtual FGs accounted for 55.7% of all identified codes. No differences were observed in the mean number of codes between virtual and in-person FGs (319 ± 6.1 vs. 254.7 ± 103.6, *p* = 0.34) (Fig. 1).

## Discussion

Herein we report findings comparing qualitative data collected via two different formats: in-person versus virtual FGs. Comparing qualitative formats requires that a comparable, rigorous approach was used in both groups to enable comparability. In the present study 95% of all excerpts and 97% of all codes (i.e., themes/sub-themes) were identified by two independent coders suggesting high fidelity and inter-coder reliability of the independent coding. Further, the coding was validated by two additional investigators (i.e., an experienced qualitative researcher, patient leader) who served as external reviewers and no additional themes/sub-themes were identified, thereby supporting that data saturation was reached in both FG formats.

Several findings support the validity of using a virtual format in lieu of in-person format for rare disease research. No differences were observed between virtual and in-person FGs in terms of number of excerpts, number of codes, or presence of codes. It is worthwhile to note that while the number of excerpts and codes were normally distributed (enabling use of Student’s T-test), the power was low (0.061, 0.10) and thus interpretation merits caution. Interestingly, as shown in Fig. 1, virtual FGs seemed to yield more consistent results as demonstrated by smaller ranges for excerpts (range 96–110 vs. 53–113) and codes (range 315–326 vs. 188–374). Moreover, examining the specific themes/sub-themes revealed that the virtual focus groups were richer in capturing personal, sensitive discussions. The in-person FGs included significantly more discussion of less personal objective topics (i.e., collapsed theme: ‘ethical concerns’ and sub-theme ‘lack of return of results’). In contrast, virtual FGs had significantly more coding related to personal/sensitive experiences (i.e., collapsed code ‘attitudes and beliefs’, ‘information and support’, ‘family communication’). Thus, while broadly comparable (i.e., number of excerpts, number of codes, code presence), the relative anonymity of virtual FGs may have helped facilitate participants sharing of experiences that were sensitive and private in nature.

Prior studies in other patient populations have highlighted the utility of virtual focus groups for engaging difficult to reach populations (i.e., adolescents) and traditionally marginalized groups (i.e., sex and gender minorities) as well as for collecting data on sensitive topics like sexual health. Among the earliest of examples, a 2008 study examined in-person and internet focus groups for qualitatively exploring relationships of gay men [28]. Investigators concluded that using the internet for qualitative research can enhance sample recruitment and produce trustworthy data. Subsequently, a 2014 study examined how online FGs affected sexual health attitudes/behavior intentions among 75 adolescent gay, bisexual, and queer males. Investigators observed positive effects with participants reporting greater comfort in talking about sex/sexuality and the online format helped them feel less isolated. Additional studies have used virtual FGs for HIV prevention in adolescents [29], examining attitudes towards human papilloma virus vaccine [30], and for eliciting perspectives and preferences for a mobile health tool for men who have sex with men [31].

Cumulatively, prior studies support the position that a virtual format can help overcome barriers to participation for hard to reach and marginalized groups and that participants feel safe and anonymous in discussing intimate topics. Rare disease patients are also hard to reach, overlooked, and patients may feel marginalized by feelings of guilt and shame that accompany a genetic/rare disease diagnosis [27, 32]. Thus, in light of our present findings, it appears that the same aspects that make web-enabled technologies useful for difficult to reach and marginalized sex/gender minorities also hold true for virtual FG discussions with rare disease patients. In other words, virtual focus groups with rare disease patients can reliably be used to complement (or replace) traditional in-person focus groups that are challenging due to geographic barriers. Rare disease patients frequently use the internet use to find information on their condition and obtain peer-to-peer support [15]. Several studies published over the past decade highlight the important role of internet and social media for patients and families living with rare diseases [27, 33–36]. Moreover, the European Reference Network on Rare Endocrine Conditions underscores the importance of effective partnerships with patient organizations for conducting needs assessments [19, 20, 27, 37]. A key aspect of understanding the unmet needs of rare disease patients involves gaining a deeper understanding of their “lived experience” through qualitative methodology [27, 38]. In the present study we partnered with a patient organization to organize FGs and leveraged web-based technologies to conduct virtual FGs.

It is worthwhile to note that this study has several limitations. First, the sample size (n = 58) is rather limited. However, from the standpoint of qualitative inquiry, the sample size is rather sizeable. Indeed, 58 participants is quite robust for qualitative research in a rare disease population. Second, participants were not matched between groups and more female patients participated in the virtual FG discussions. This observation is due to the request for a “women’s only” meeting during the conduct of virtual FGs. A central aspect of qualitative research is the concept of saturation. Saturation refers to the time at which no new codes emerge. Traditionally saturation is reached by conducting three to five focus group discussions [26]. We have previously shown that saturation can be reached in three focus groups when probing a specific topic with rare disease patients [27]. In the present study we conducted three in-person and three virtual FGs enabling us to reach data saturation and appropriate comparison between methods—as confirmed by external reviewers. Another consideration for the study is that we used statistical methods to compare in-person and virtual FGs. While some forms of qualitative research ‘quantitize’ qualitative findings, qualitative inquiry does not traditionally rely on quantitative analysis. Rather, the goal of qualitative inquiry to gain a deeper understanding of patients’ lived experiences and perspectives. As such, this validation study may veer from traditional views of qualitative research. The rationale for this approach was to support the methodologic validity and rigor of using web-enabled technologies (i.e. virtual FGs) for conducting qualitative research in a rare disease population.

## Conclusions

Geographically dispersed patients have posed significant roadblocks for rare disease research resulting in small sample sizes and underpowered studies. As rare disease patients have been referred to as internet “power users”, web-enabled technologies hold promise for reaching dispersed rare disease patients and surmounting geographic barriers for many. To our knowledge, the present study is the first to support the validity of virtual focus groups for qualitative rare disease research. Moreover, the present findings suggest the anonymity afforded by the internet can facilitate discussion of highly sensitive and intimate topics. This observation is important as feelings of stigma and shame are frequently experienced by patients living with a rare disease—particularly in a condition like CHH that has a psychosexual/sexual health component [19–22]. The present findings support methodologic rigor of using web-enabled technologies for qualitative research using focus groups in rare diseases.

## Supplementary Information

**Additional file 1.** Focus group questions/prompts.

**Additional file 2.** Presence of themes and sub-themes by focus group.

## Data Availability

De-identified data will be made readily available upon request for research purposes to qualified individuals within the scientific community.

## Abbreviations

CHH: Congenital hypogonadotropic hypogonadism
FG: Focus group
GnRH: Gonadotropin releasing hormone
KS: Kallmann syndrome
SD: Standard deviation
