# Supplementary material for: Advancing qualitative rare disease research methodology: a comparison of virtual and in-person focus group formats
Source: Orphanet J Rare Dis. 2022 Sep 11;17:354. doi: 10.1186/s13023-022-02522-3 (PMC9465872; doi:10.1186/s13023-022-02522-3)
Supplement: Supplementary file 1 — Additional file 1. Focus group questions/prompts. [file 13023_2022_2522_MOESM1_ESM.docx]

**Additional file 1.** Focus group questions/prompts

We would like to better understand your thoughts and experiences regarding genetic testing. We are interested in hearing your opinion, there are no right or wrong answers.

- As a patient or parent/guardian, what are the things that you consider to be important for making a decision whether to have genetic testing or not?
- Where did you get information that helped you make a decision about genetic testing?
- What do you think the needs are for information and support around decisions for genetic testing?
- CHH is heritable, meaning it runs in families. Did you speak with family members about potential risk? What prevented you from doing so and was there anything that was helpful in doing this?
