# Supplementary material for: Advancing qualitative rare disease research methodology: a comparison of virtual and in-person focus group formats
Source: Orphanet J Rare Dis. 2022 Sep 11;17:354. doi: 10.1186/s13023-022-02522-3 (PMC9465872; doi:10.1186/s13023-022-02522-3)
Supplement: Supplementary file 2 — Additional file 2. Presence of themes and sub-themes by focus group. [file 13023_2022_2522_MOESM2_ESM.docx]

**Additional file 2**. Presence of themes and sub-themes by focus group

| **Theme •** Sub-theme | **In-person** | | | **Virtual** | | |
| --- | --- | --- | --- | --- | --- | --- |
|  | **# 1** | **# 2** | **# 3** | **# 1** | **# 2** | **# 3** |
| **Attitudes & Beliefs**  • motivating factors  • test type  • uncertainty  • cost of testing | yes  yes  yes  yes  yes | yes  yes  yes  yes  **NO** | yes  yes  yes  yes  yes | yes  yes  yes  yes  yes | yes  yes  yes  yes  yes | yes  yes  yes  yes  yes |
| **Information & Support**  • information source  • pre-test decision support  • genetic counseling | yes  yes  yes  yes | yes  yes  yes  yes | yes  yes  yes  yes | yes  yes  yes  yes | yes  yes  yes  yes | yes  yes  yes  yes |
| **Return of Results**  • uncertainty  • results interpretation  • lack of results, waiting  • lack of post-test support | yes  yes  yes  yes  yes | yes  yes  yes  yes  yes | yes  yes  yes  yes  yes | yes  yes  yes  yes  yes | yes  yes  yes  yes  yes | yes  yes  yes  yes  yes |
| **Family Communication**  • barriers  • promoters | yes  yes  yes | yes  yes  yes | yes  yes  yes | yes  yes  yes | yes  yes  yes | yes  yes  yes |
| **Ethical Concerns**  • privacy & data use  • sample traceability  • informed consent | yes  yes  yes  yes | yes  yes  yes  yes | yes  yes  **NO**  **NO** | yes  yes  **NO**  **NO** | yes  yes  **NO**  yes | yes  yes  yes  yes |
